# Impact of a Three-Strain Lactobacilli Probiotic (BioK+) on Incidence of Hospital-Onset *Clostridioides difficile*: A Retrospective Observational Cohort Study

**DOI:** 10.3390/antibiotics14121225

**Published:** 2025-12-04

**Authors:** Matthew A. Jenest, Randolph V. Fugit, Jason Wright, Mary T. Bessesen, Shelley E. Kon

**Affiliations:** 1 VA Eastern Colorado Health Care System, 1700 N. Wheeling St., Aurora, CO 80045, USA; matthew.jenest@va.gov (M.A.J.); randolph.fugit@va.gov (R.V.F.); mary.bessesen@va.gov (M.T.B.); 2 Department of Pharmacy Practice, Skaggs School of Pharmacy & Pharmaceutical Sciences, Anschutz Campus, 12850 East Montview Boulevard, Aurora, CO 80045, USA; 3 Department of Medicine, Division of Infectious Diseases, Anschutz Campus, University of Colorado School of Medicine, 13001 E 17th Pl, Aurora, CO 80045, USA

**Keywords:** probiotics, infection control, hospital-acquired infections, *Clostridioides difficile*, HO-CDI, BioK+

## Abstract

**Background:** Prevention of hospital-onset *Clostridioides difficile* infection (HO-CDI) is a priority for hospitals. In addition to standard infection control measures, some probiotics show promise in reducing HO-CDI incidence. However, prior research has produced mixed results. **Methods:** Retrospective, observational cohort study of HO-CDI incidence among inpatients treated with or without BioK+ probiotic prophylaxis. BioK+, a probiotic with three *Lactobacilli* strains, was administered to patients on antibiotics with high risk for HO-CDI. BioK+ was continued for 5 days after antibiotics were discontinued, or the patient was discharged. The primary outcome was HO-CDI incidence. **Results:** Out of 494 eligible patients on high-risk antibiotics, 343 patients received BioK+ probiotics. No cases of HO-CDI were identified in patients who received BioK+, compared to three cases among patients not on BioK+ (*p* = 0.028). In the baseline period (1 April 2021–31 March 2022) the HO-CDI incidence density was 5.62 per 10,000 bed-days. In the BioK+ probiotic period (1 April 2022–31 March 2023), the incidence density was 2.22 cases per 10,000 patient days (*p* = 0.03). **Conclusions:** When bundled with standard infection control practices, the use of BioK+ probiotics was associated with a statistically significant decreased incidence of HO-CDI among patients prescribed high-risk antibiotics.

## 1. Introduction

*Clostridioides difficile* infection (CDI) can cause mild to severe diarrhea, abdominal pain, fever and potentially sepsis and life-threatening colitis. The United States (US) Centers for Disease Control and Prevention (CDC) has classified *C. difficile* as an urgent threat due to the excess morbidity, mortality and significant healthcare expenditures associated with CDI [1]. Although healthcare-associated CDI cases have declined in recent years, it remains one of the most common healthcare-associated infections in the US [2]. One in 11 patients over 65 who acquire CDI in the hospital will die within 30 days, creating a pressing need to explore new ways to reduce hospital-onset HO-CDI [1].

Antibiotic use is one of the leading risk factors for CDI, present in 60% of cases [1]. Antibiotic treatment alters the natural microbiome of the gastrointestinal tract, increasing the risk for *C. difficile* to overgrow in the intestines [1]. Probiotics have been proposed as an intervention to reduce the risk of CDI, as they offer a theoretical benefit in preserving the natural microbiome [3]. Prior studies have explored their effect on antibiotic-associated diarrhea and hospital-onset CDI. For example, a recent multicenter, randomized, double-blind, placebo-controlled trial demonstrated that a high-dose multi-strain probiotic mix was effective at reducing antibiotic-associated diarrhea (AAD) by 64% (9.2 vs. 25.3%, *p* = 0.001) [4]. A meta-analysis of 31 randomized controlled trials, which included 8672 patients, concluded that probiotics are effective for preventing CDI [5]. However, this and other meta-analyses have included heterogeneous studies that utilized different probiotic species and took place in different care settings [5,6]. The US Food and Drug Administration (FDA) has not approved any probiotic as a live biotherapeutic product with an indication for *C. difficile* infection prevention [7]. The Society for Healthcare Epidemiology of America (SHEA) considers the use of probiotics for primary prophylaxis of HO-CDI to be an unresolved issue [2].

At our Veterans Administration (VA) medical center, all of the SHEA essential practices for preventing HO-CDI in acute-care hospitals were in place [2]. However, we continued to see an elevated incidence of HO-CDI. Therefore, our infection prevention and control (IPC) team explored additional approaches to reduce HO-CDI rates. A multidisciplinary team selected BioK+, which had supporting efficacy data from randomized controlled clinical trials and is approved by Health Canada for the prevention of CDI [5,8,9,10].

BioK+ is composed of *Lacticaseibacillus casei* LBC80R^®^, *Lacticaseibacillus rhamnosus* CLR2^®^, and *Lactobacillus acidophilus* CL1285^®^. A meta-analysis of three randomized controlled trials demonstrated a significant reduction in CDI risk with this probiotic formula compared with placebo (RR = 0.21; 95% CI: 0.11–0.40) [11]. However, real-world implementation of this specific probiotic has produced mixed results, with three studies showing lower HO-CDI incidence [12,13,14] and three negative studies [15,16,17]. One of the three positive studies examined probiotics as part of a bundle of preventative measures [14]. Additional research on the effect of probiotic therapy for patients treated with high-risk antibiotics is warranted given the mixed results of previous research. We conducted a retrospective observational cohort study to compare the incidence of HO-CDI among patients treated with BioK+ with that among patients who did not receive probiotics. In addition, the facility-wide HO-CDI rates were compared pre/post intervention periods.

## 2. Results

### 2.1. Patient-Level

**Figure 1:**
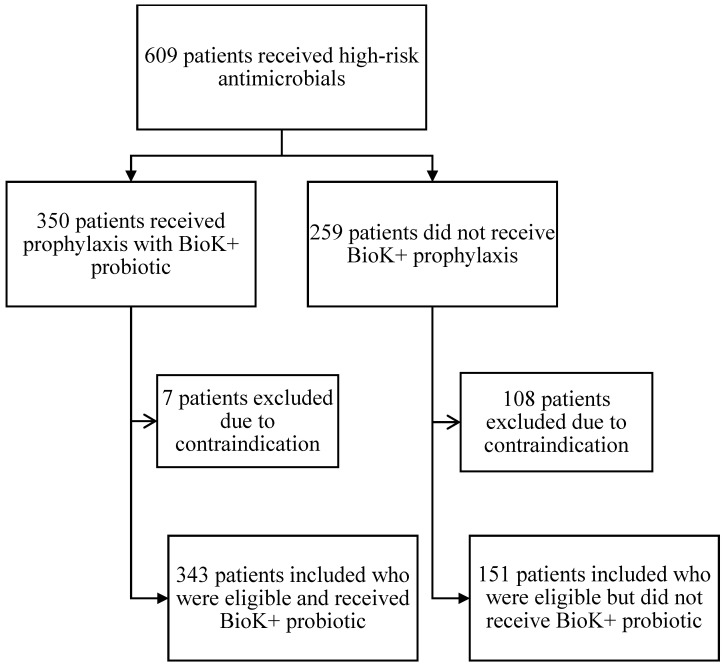
Flow chart for screening, evaluation, and analysis.

A total of 609 inpatients receiving high-risk antibiotics during the intervention period were screened (Figure 1). Of the 350 patients who received BioK+ prophylaxis, 7 were excluded from the analysis due to impaired intestinal integrity (4 patients), CDI present at admission (2 patients), and immunocompromising condition (1 patient with a history of organ transplantation). A final total of 343 patients who received one or more doses of BioK+ were analyzed.

An additional 259 patients were identified as having received high-risk antimicrobials but not the BioK+ probiotic. Of these 259, 108 patients were excluded due to impaired intestinal integrity (85 patients), immunocompromised status (6 patients with a history of organ transplant, 4 patients with a history of hematologic malignancy, 1 patient with ANC <500 cells/µL and 1 patient with a history of HIV and CD4 <200 cells/µL), CDI in the past 60 days prior to admission (2 patients) and active community-associated *C. difficile* infection (CA-CDI) on admission (9 patients), leaving 151 eligible patients who did not receive BioK+. Overall adherence to the pathway’s inclusion and exclusion criteria was 69% (343 of the total 494 eligible patients received BioK+).

The demographics and baseline characteristics of the entire patient population are described in Table 1a. There were no statistically significant differences between groups in terms of age, sex assigned at birth, race, prior CDI, PPI use, tube feeding or length of stay. The proportion of patients admitted to the ICU was significantly higher in the non-treatment group than in those treated with BioK+ (*p* = 0.03). Due to the imbalance in ICU cases in the treatment and control groups, an analysis limited to patients who were not in the ICU was performed and showed no cases of HO-CDI among the 312 patients on BioK+ and three cases among the 127 eligible patients who were not treated (*p* = 0.03). The baseline characteristics of the floor-status patients are described in Table 1b.

**Figure 2:**
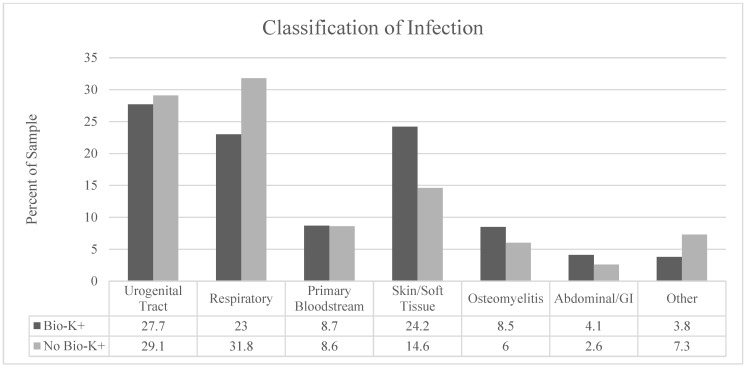
Classification of the types of infections in each group by anatomic site.

**Figure 3:**
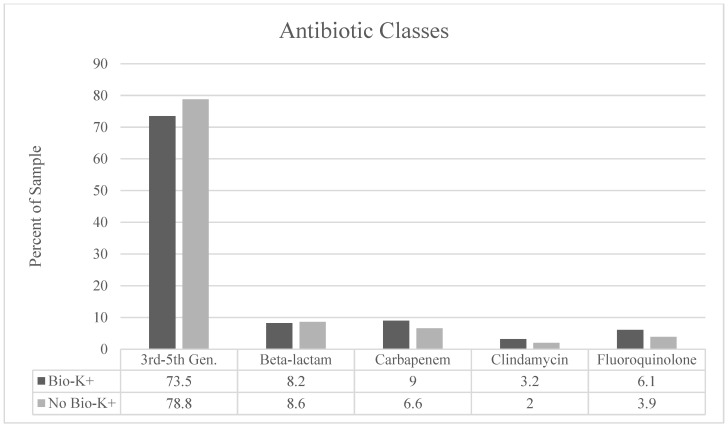
Classification of antibiotics used by antimicrobial class. There were no statistically significant differences in antibiotics by class between the patients who received BioK+ and those who did not receive BioK+.

Infection by type and antibiotic use by class are described in Figure 2 and Figure 3, respectively. We observed a significantly higher proportion of patients treated for skin/soft tissue in the treatment group (*p* = 0.02) and a larger proportion of respiratory infections in the non-treatment group (*p* = 0.04). Third- to fifth-generation cephalosporins were the most prescribed antibiotic class. There were no statistically significant differences between the groups in terms of antibiotic selection. BioK+ prophylaxis was prescribed appropriately and per hospital pathway in 98% of cases that received BioK+.

There were no cases of HO-CDI among the 343 eligible patients who received BioK+, while three HO-CDI cases occurred among the 151 eligible patients who did not receive BioK+ prophylaxis (*p* = 0.028) (Table 2). All HO-CDI cases observed in the non-treatment group had symptomatic diarrhea and were non-severe.

Three patients were treated with oral vancomycin prophylaxis while taking high-risk antibiotics. All were in the group that did not receive BioK+; none of them developed CDI. The average time from the first order for a high-risk antibiotic to the order for BioK+ prophylaxis was 36.6 h. Most (86.6%) eligible patients who were treated with BioK+ received their first dose within 72 h of the first dose of high-risk antibiotic. The median duration of the BioK+ treatment was 5 days (interquartile range, 3 to 9 days).

No cases of lactobacillus bacteremia were observed among patients who received BioK+, including those patients who were ineligible but received BioK+.

### 2.2. Facility Level

In the baseline period (1 April 2021–31 March 2022) the incidence density of HO-CDI was 5.62 per 10,000 patient days (19 cases in 33,787 patient days). In the BioK+ period (1 April 2022–31 March 2023) the incidence density was 2.22 cases per 10,000 patient days (7 cases in 31,563 patient days (*p* = 0.03). Four additional cases of HO-CDI (one severe and three non-severe) were identified among patients who did not receive BioK+ because they were ineligible based on our pathway.

## 3. Discussion

Patients on high-risk antibiotics who received BioK+ had a statistically significant lower incidence of HO-CDI than patients who did not receive BioK+. Pathway adherence was high, with BioK+ being prescribed to 69% of eligible patients at an average start time of 36.6 h. The use of BioK+ proved to be safe. No cases of Lactobacillus bacteremia were observed in treated patients, including in the seven patients who inadvertently received BioK+ despite meeting exclusion criteria (Figure 1). This is consistent with the safety outcomes observed in other studies [5].

In addition, the facility-wide HO-CDI incidence density improved significantly during the BioK+ treatment period compared to the incidence prior to the study implementation. This result is consistent with three previous observational studies that showed an association between prophylaxis with BioK+ and reduced incidence of *Clostridioides difficile* disease [12,13,14]. Two prospective observational studies of BioK+ performed in a large Canadian hospital system showed large reductions in *C. difficile* incidence associated with introduction of prophylaxis with BioK+ [12,13]. Use of BioK+ as part of a bundle of interventions in a high-risk unit of a hospital in Florida was associated with a decrease in HO-CDI incidence density from 14.7/10,000 patient days to 3.12/10,000 patient days [14]. Three observational studies did not show a reduced incidence of HO-CDI when the same probiotic, BioK+, was used [15,16,17]. Box et al. found CDI incidence was 1.8% among patients on antibiotics who were given prophylaxis with BioK+ versus 0.9% among patients who were not given BioK+ (*p* = 0.16) [15]. The patients given BioK+ prophylaxis had significantly greater antibiotic exposure, higher Charlson comorbidity scores, and longer lengths of stay, which may have contributed to the higher incidence of HO-CDI in the treated group in that study. Heil et al. found a statistically significant increase in the odds of CDI among patients given BioK+ prophylaxis in a multicenter propensity score-matched observational study [16]. Adherence to the probiotic pathway among eligible patients in the studies by Box and Heil was 41–46%, lower than in the present study. In a cluster-randomized trial, Leal et al. found a decrease in HO-CDI; however, it was not statistically significant [17]. In the Leal study there was a lower baseline CDI incidence (ranging from approximately 2–4 per 10,000 patient days), patients on all antibiotics were included and the probiotic was started later than 72 h after antibiotic therapy in 24.2% to 33.3% of cases. In contrast, our study included patients on high-risk antibiotics, our facility had a higher baseline HO-CDI incidence, and patients were started promptly after antibiotic administration. These key differences may explain why our study found a significant reduction in CDI rates.

Our study has several limitations. First, this study was a single-center, non-randomized, retrospective study. With the patient-level study, there were some imbalances between the groups, and we did not match treated patients with controls. One notable imbalance was that the proportion of patients admitted to the ICU was significantly higher among patients who did not receive BioK+. To account for this difference in patient characteristics, we conducted an additional statistical review on only the patients that were admitted to medicine floors outside of the ICU. There were no significant baseline differences between treated and untreated floor-status patients. While this imbalance is important to acknowledge, it ultimately did not have a significant impact on the results of this study, given that none of the cases of HO-CDI occurred among ICU patients. When ICU cases were excluded from the analysis, the results showing a lower risk of HO-CDI for patients given BioK+ remained statistically significant. The lower enrollment of eligible patients in the ICU was not caused by exclusion criteria. We hypothesize that this may be due to the ICU provider’s hesitance to use probiotics in critically ill patients; however, this area needs further research. Additional statistical differences included a higher proportion of treated patients with skin/soft tissue infections and a higher proportion of non-treated patients with respiratory infections. Patients were not in different medical wards based on infection type, and the provider pool was the same for both groups, so it is unclear why there was an imbalance. We hypothesize that this may be due to the perception that patients with skin/soft tissue infection will require longer durations of antibiotic therapy, despite recommendations for the majority of patients to be on 5–7 days of antimicrobials. However, this information was not collected and would need further study. If perhaps these patients were at higher risk, this would have biased the study toward the null hypothesis, as they would be more likely to be diagnosed with HO-CDI, obscuring the effect of BioK+. There may be additional unmeasured differences between the treated and untreated patient groups. It is possible that the presence of a prescription for CDI prophylaxis in a patient’s chart may lead to heightened infection control measures, since the patients are now identified as high-risk to staff. Our retrospective design relied on chart documentation for our study data. While chart review has the potential to be subjective, our largely objective data points reduced observational bias. Additionally, our veteran population is heavily comprised of elderly white males, which might impact the generalizability of these results. The facility-level pre/post comparison is limited by multiple interventions taking place in the baseline period prior to implementation of the BioK+ pathway. However, both treated and untreated patients experienced equivalent infection control interventions; therefore, this should not have affected the patient-level comparison. It is unknown if changes in the frequency of *C. difficile* testing occurred during the two study periods, as this data was not collected. Overall, it is important to note that the implementation of the BioK+ pathway was a part of a bundle approach. Multiple interventions occurred in the baseline period and must be considered; however, the significant and sustained reduction in incidence of HO-CDI after the initiation of this pathway suggests that this was an important individual intervention. Although a generic probiotic was on formulary, no patients received the generic probiotic and BioK+ simultaneously during the study period. Other seasonal trends of HO-CDI were likely minimized, as the two study periods were done for the same monthly time periods (April–March). Lastly, as this study was not a randomized, controlled trial, our rates may have been confounded by factors we did not control for, thus future clinical trials are warranted to explore the effect of BioK+ on HO-CDI incidence.

## 4. Materials and Methods

### 4.1. Study Design

We performed a retrospective, observational cohort study of HO-CDI incidence among inpatients who received high-risk antibiotics with or without BioK+ between 1 April 2022 and 31 March 2023. We also compared facility-level HO-CDI rates during the intervention year and compared the rates to a pre-intervention year (April 2021–March 2022).

### 4.2. Setting

The study took place at a 180-bed academically affiliated Veterans Affairs (VA) Hospital in Aurora, CO, USA.

### 4.3. Antibiotic Stewardship Program

The hospital had a robust antibiotic stewardship program, which included clinical pathways for common infections that recommended the narrowest spectrum agents appropriate to the diagnosis. Concurrent review and feedback of inpatient antimicrobial therapy was conducted daily on weekdays by an infectious diseases pharmacist.

### 4.4. Baseline Infection Prevention and Control Practices

Infection Preventionists performed routine surveillance for HO-CDI utilizing NHSN criteria, which defines HO-CDI as a positive *C. difficile* lab test ≥4 days after healthcare facility admission, with the day of admission counted as day 1. Recurrent infections were excluded from the HO-CDI rate per NHSN guidelines [2]. Laboratory testing for *C. difficile* was performed by Cepheid PCR (© 2024 Cepheid^®^, Sunnyvale, CA, USA and other countries). Prior to the implementation of BioK+ prophylaxis, all the SHEA essential practices to prevent HO-CDI were in place, including annual IPC training for staff and hand hygiene surveillance. Clinicians had access to a decision tree for *C. difficile* testing. The decision tree advised only testing patients for *C. difficile* if patients had 3 or more unexplained watery stools in the last 24 h, were not on laxatives or previously positive for *C. difficile* in the last 7 days. In March 2022, a best-practice advisory was built in the electronic medical record to guide clinicians to defer testing for CDI in patients taking laxatives and other medications associated with diarrhea.

Environmental Management Services (EMS) utilized checklists, sporicidal agents and ATP monitoring for evaluation of room cleaning. The EMS checklist ensures two-step cleaning and sporicidal disinfection of high-touch surfaces and is signed off by a supervisor. In August 2021 the Food and Nutrition service began using disposable food trays for all patients in contact plus precautions for suspected or known CDI.

### 4.5. Intervention

In April 2022 our facility implemented a pathway to prescribe a defined probiotic composed of *Lacticaseibacillus casei* LBC80R^®^, *Lacticaseibacillus rhamnosus* CLR2^®^, and *Lactobacillus acidophilus* CL1285^®^ (BioK+) to eligible patients at increased risk of HO-CDI. BioK+, equivalent to 100 billion colony-forming units (CFU), was administered as either 2 capsules containing 50 billion CFU each daily for patients taking food or medication by mouth, or via a liquid formulation for patients with feeding tubes. BioK+ was continued for the duration of high-risk antibiotic use plus five days or until discharge, whichever came first. The pathway was implemented hospital-wide. Use of the pathways was promoted by education given to providers and pharmacists, distribution of an educational flyer and discussion at huddles but was ultimately at the discretion of the treating team.

### 4.6. Inclusion and Exclusion Criteria

Eligibility criteria included inpatient status and a new prescription for at least one of the following antimicrobials with an expected duration of ≥48 h: 3rd–5th generation cephalosporin, fluoroquinolone, carbapenem, beta-lactam/beta-lactamase inhibitor, clindamycin or aztreonam. These antibiotics were chosen as they have been associated with a higher risk for CDI compared to other antibiotics [18]. In accordance with the pathway, providers and pharmacists were encouraged to screen patients and prescribe BioK+ to eligible patients. The initiation of the study probiotic was encouraged as soon as the patient met eligibility criteria; however, there was no requirement to initiate within a certain timeframe. Although encouraged, the decision to utilize the pathway was left to the discretion of the treating provider. Clinical pharmacists were given privileges to initiate therapy in appropriate patients under their scope of practice. A generic probiotic, *Lactobacillus acidophilus* (Nature’s Blend^®^ Dietary supplement), was available at our facility, but patients were not recommended to be on both probiotic products simultaneously. Exclusion criteria were neutropenia with absolute neutrophil count <500 cells/µL, active hematologic malignancy, history of hematopoietic stem cell or solid organ transplant, HIV with CD4 count <200 cells/µL, CDI within the past 60 days, pregnancy or impaired intestinal integrity (acute pancreatitis, bowel surgery within four weeks, colitis within two weeks, bowel obstruction, ileus). Patients who received BioK+ with antibiotics other than those specified by our pathway were excluded. Patients in both groups were excluded if CDI was present on admission. At the provider’s discretion, patients with a previous history of CDI could be treated with prophylactic oral vancomycin when receiving high-risk antibiotics.

### 4.7. Data Source

Patients were identified using VA electronic health records. Demographic factors were self-reported in the medical record. Patients who received probiotic prophylaxis were identified via a report of all the BioK+ prescriptions during the study period. An additional report was run to identify all prescriptions for high-risk antibiotics during the study period. Severe CDI cases were defined as those with WBC count ≥15 × 10^3^ cells/µL or serum creatinine ≥1.5 mg/dL on the day of the positive *C. difficile* test [19].

### 4.8. Patient Consent Statement

The study pathway and waiver of consent were approved by the Colorado Multiple Institutional Review Board and by the VA Eastern Colorado Healthcare System Research and Development Committee (Protocol #22-1793).

### 4.9. Outcome Measures

The primary outcome was patient-level incidence of HO-CDI, as defined above and by the National Healthcare Safety Network (NHSN) [2]. Patients who received BioK+ were compared to patients who were eligible but did not receive BioK+ during the study intervention period (April 2022–March 2023). Facility-level HO-CDI incidence was compared pre-study (April 2021–March 2022) and during the study intervention period (April 2022–March 2023).

The secondary safety outcome was the incidence of *Lactobacillus* bacteremia. We also determined adherence to the treatment pathway based on percentage of eligible patients who received BioK+ over the total number of eligible patients. Finally, we identified the proportion of ineligible patients who inadvertently received BioK+.

### 4.10. Patient-Level Baseline Variables

We collected patient demographics and clinical characteristics during the time of high-risk antibiotic use. Major clinical characteristics included the type of underlying infection, the antimicrobial or class of antimicrobial prescribed, treatment duration and total length of hospital stay. History of prior CDI, ICU or floor status, tube feeding use and proton pump inhibitor (PPI) use were also recorded. For patients who did not meet pathway eligibility, the reasons for ineligibility were documented from medical records.

### 4.11. Statistical Analysis

HO-CDI incidence, defined as the number of HO-CDI cases per 10,000 patient days, was calculated for the pre- and post-intervention study periods. Statistical analysis was performed using GraphPad Prism version 9 software. A two-sided Fisher’s exact test was used to analyze categorical data. A *t*-test was used to analyze normally distributed continuous data. A Mann–Whitney test was used to analyze continuous data that were not normally distributed.

## 5. Conclusions

In our study, the addition of BioK+ to an established bundle of infection-control practices was safe and effective and associated with a reduction in the HO-CDI incidence among treated patients.

## Figures and Tables

**Table 1:** (**a**) Characteristics of all eligible patients receiving the study probiotic (BioK+) compared to patients not receiving the probiotic. (**b**) Characteristics of eligible floor-status patients receiving the study probiotic (BioK+) compared to patients not receiving the probiotic.

**(** **a)**			
**Characteristic**	**Study Probiotic BioK+** **(N = 343)**	**No BioK+ Given** **(N = 151)**	***p***
Age (Mean years ± SD)	68 (±14)	67 (±13)	0.39
Sex (% male)	93.0	92.7	NS
Race (% White)	77.8	75.5	NS
ICU (%)	9.0	15.9	0.03
Prior *C. difficile* (%)	4.1	2.6	0.6
PPI (%)	29.7	29.8	NS
BioK+ probiotic days (Mean ± SD)	7 (±9)	-	n/a
Length of Stay (Mean ± SD)	24 (±52)	14 (±31)	0.29
Total length of stay (Median, range, IQR)	7 (1–427, 4–17)	6 (2–272, 4–10)	
Tube feed	9 (2.6%)	3 (2.0%)	>0.99
**Infection type**			
GU	95 (27.7%)	44 (29.1%)	0.75
Respiratory	79 (23.0%)	48 (31.8%)	0.04
Primary bloodstream	30 (8.7%)	13 (8.6%)	>0.99
SSTI	83 (24.2%)	22 (14.6%)	0.02
OM	29 (8.5%)	9 (6.0%)	0.46
Abdominal/GI	14 (4.1%)	4 (2.6%)	0.60
Other	13 (3.8%)	11 (7.3%)	0.11
**(** **b)**			
**Characteristic**	**Study Probiotic BioK+** **(N = 312)**	**No BioK+ Given** **(N = 127)**	***p***
Age (Mean years ± SD)	68 (±14)	67 (±14)	0.45
Sex (% male)	92.6	93.7	0.84
Race (% White)	77.6	76.4	0.80
Prior *C. difficile* (%)	4.5	1.6	0.17
PPI (%)	27.6	24.4	0.63
Defined probiotic days (Mean ± SD)	7 (±9)	-	n/a
Length of Stay (Mean ± SD)	22 (±47)	14 (±33)	0.20
Total length of stay (Median, range, IQR)	7 (1–427, 4–16)	6 (2–272, 4–10)	

**Table 2:** Outcomes among patients treated with BioK+ vs. not treated.

Outcome	BioK+ Prophylaxis	No BioK+ Given	*p*
HO-CDI among concurrent eligible patients	0/343 (0%)	3/151 (2.0%)	0.028
	Prophylaxis Period 1 April 2022–31 March 2023	Baseline Period 1 April 2021–31 March 2022	
HO-CDI among all hospitalized patients (cases per 10,000 patient days)	2.22	5.62	0.03

## Data Availability

Aggregate data will be shared with researchers who submit an analysis pathway that is approved by their institutional review board and by the VA Eastern Colorado Healthcare System Research and Development Committee.
